# Fenestrating Versus Reconstituting Subtotal Cholecystectomy: Systematic Review and Meta-Analysis on Bile Leak, Bile Duct Injury, and Outcomes

**DOI:** 10.7759/cureus.72769

**Published:** 2024-10-31

**Authors:** Kapilraj Ravendran, Ahmed Elmoraly, Christo S Thomas, Mridhu L Job, Afrah A Vahab, Shafali Khanom, Chloe Kam

**Affiliations:** 1 Surgery, Royal National Orthopaedic Hospital, Brighton and Hove, GBR; 2 Doctor, Gradscape, London, GBR; 3 General Medicine, East Sussex Healthcare NHS Trust, Hastings, GBR; 4 Surgery, Medical University of Varna, Varna, BGR; 5 Medicine, Gradscape, London, GBR; 6 Surgery, Medical University Sofia, Sofia, BGR; 7 Endocrinology, Medical University of Sofia, Sofia, BGR

**Keywords:** bile leak, fenestrated, lap cholecystectomy, post cholecystectomy bile duct injury, reconstituted, subtotal

## Abstract

Symptoms of gallstone disease are the most common reason for cholecystectomy. Fenestration reduces the likelihood of severe inflammation or scarring after normal treatments are used, and it also enhances control over bile outflow. The goal of reconstituted cholecystectomy is to lessen symptoms like pain and jaundice without undergoing the high-risk procedures associated with more invasive procedures. The reconstituted and fenestrated procedures were assessed by a meta-analysis and systematic review. Of the five studies, 189 (34.2%) had a reconstituted subtotal cholecystectomy, and 363 (65.8%) had a fenestrated subtotal cholecystectomy, which had populations from the United States of America, the United Kingdom, Japan, and Turkey. Two individuals from three trials had bile duct injury, according to three studies. Whereas the fenestrated group reported no bile injury from 236 individuals (0%), the reconstituted group reported two bile duct injuries from 100 patients (2%). The incidence was found to be lower in the fenestrated group (OR 10.81; CI 95% 1.03-113.65; p = 0.39; I2 = 0%) than in the reconstituted group. Four studies revealed 92 cases of bile leaks: 19 out of 155 cases (12.3%) were reconstituted, and 73 out of 351 cases (20.8%) were fenestrated. Between the two groups, there was a significant difference in bile leakage (OR 0.72; CI 95% 0.23-2.32; p = 0.03; I2 = 66%). Two studies reported the establishment of fistulas following surgery in 58 patients in the reconstituted group (5.2%) and 120 patients in the fenestrated group (2.5%) (p = 0.56, I2 = 0%, and OR 0.65; CI 95% 0.12-3.38); however, there was no statistically significant difference between the groups. Following a fenestrated partial cholecystectomy, postoperative bile leakage, fistula development, wound infection, and retained stones are more prevalent. Additionally, we saw that the fenestrated method was being used more frequently for post-operative endoscopic retrograde cholangiopancreatography (ERCP). The subtotal cholecystectomy technique used should be chosen according to the surgeon's comfort level and experience with the various techniques and intraoperative findings, even if the reconstituted procedure could be preferred when feasible. To completely understand the role of each method in the general surgeon's toolkit for treating complex gallbladder (GB) patients, longer-term follow-up studies are still necessary.

## Introduction and background

Gallstones affect about 15% of adults in the UK; most have no symptoms, with only 2-4% experiencing symptoms and aftereffects [1,2]. The most frequent cause of cholecystectomy is gallstone disease symptoms. Rarely, cholecystectomy may be performed for gallbladder (GB) polyps or cancer [1]. Since its inception in the late 1980s, laparoscopic cholecystectomy (LC) has supplanted open operation as the gold standard, mainly because of a shorter hospital stay and a reduced risk of complications [1,3,4]. On the other hand, LC is linked to higher rates of bile duct damage (BDI) than open procedures [5-7]. The deformed architecture of the Calot's triangle can be caused by complicating conditions such as fibrosis, gangrenous cholecystitis, gangrenous empyema, intra-abdominal adhesions, and Mirizzi's syndrome [8]. By mobilizing the bottom third of the GB and verifying that the cystic duct and artery are the only two structures linked to it, the critical view of safety (CVS) seeks to accurately identify the GB [9]. In cases where the CVS was unavailable, an open conversion was often carried out to facilitate cautious dissection and lower the possibility of BDI [10].

When a laparoscopic cholecystectomy proves problematic because the crucial view of safety is not reached, the anatomical structures implicated are not identified adequately, or there is a danger of harm, a subtotal cholecystectomy is performed as a last resort [11]. The 2020 World Journal of Emergency Surgery recommends subtotal cholecystectomy for acute calculous cholecystitis when identifying the necessary anatomical structures is difficult or there is a high risk of an iatrogenic injury [12]. When a difficult cholecystectomy prevents a patient from reaching the crucial perspective of safety, subtotal cholecystectomy is considered the best bailout method [13]. With higher success rates than ever before, subtotal cholecystectomy is a technique that has only grown in importance over time [13].

A variant of subtotal cholecystectomy is fenestrated cholecystectomy [14]. Fenestrating refers to the removal of the free, peritonealized gallbladder from a lip at the lowest part of the gallbladder without the formation of a gallbladder residue [11]. This serves as a barrier to prevent unintentionally entering the hepatocystic triangle (also known as the "Shield" of McElmoyle) [11]. The liver leaves the gallbladder component in place [11]. The gallbladder's sliced edge may be oversewn after removing the stones [11]. A purse-string suture (inset) may be used to seal the cystic duct from the inside after the mucosa has been ablated [11]. Attempts to ligate the cystic duct outside the gallbladder may harm the common bile duct due to its potential short length [11]. In order to improve bile drainage, a portion of the gallbladder wall must be purposely opened [14]. This method is particularly useful in challenging situations where there is a lot of adhesion or fibrosis around the gallbladder [14]. It lessens the chance of bile leakage and enables improved control over bile flow [14]. When utilizing standard techniques with a high risk of scarring or severe inflammation, fenestration helps minimize postoperative problems and improves control of bile outflow [14].

On the other hand, cholecystostomy (making an incision and removing the gallstone) entails the establishment of a permanent drainage tube between the gallbladder and the exterior of the gastrointestinal system [15]. When the gallbladder is highly infected or inflammatory, total removal becomes too risky [15]. Reducing symptoms like pain and jaundice is the aim of reconstituted cholecystectomy, while avoiding the high-risk consequences linked to more intrusive operations [15]. Reconstituting (generating a gallbladder remnant) removes the free, peritonealized part of the gallbladder [11]. The segment of the gallbladder that is attached to the liver can either be partly removed or left in place [11]. Only the lowest section of the gallbladder is left when the lowest piece is sutured or stapled shut, creating an intact lumen where stones may re-form [11]. Restoring an intact lumen may cause stones to reappear [11]. Whether a subtotal cholecystectomy is "fenestrating" or "reconstituting" depends on whether the lowest portion of the gallbladder remains open (fenestrating) or closed (reconstituting) [11].

Since few studies contrast the two variations of laparoscopic subtotal cholecystectomy, fenestrated and reconstituted subtotal cholecystectomy, a meta-analysis and systematic review were carried out to evaluate the reconstituted and fenestrated methods.

## Review

Methodology

Search Strategy

All authors collaboratively designed and agreed on the protocol, and followed the PRISMA (Preferred Reporting Items for Systematic Reviews and Meta-analyses) standards in the conduct of this research [16]. The review adhered to the PRISMA principles [16]. Bias risk was assessed, and disagreements on bias and the best way to interpret the data were resolved by consensus-building talks.

A literature search was performed via MEDLINE (available through PubMed), Cochrane (including the Cochrane Database of Systematic Reviews, Cochrane Central Register of Controlled Trials, Cochrane Methodology Register, Database of Abstracts of Reviews of Effects, and National Health Service Economic Evaluation Database), MEDLINE, EMBASE, and Google Scholar using the following set of keywords: “subtotal” OR “partial” OR “incomplete” OR “cholecystectomy” AND “fenestrating” OR “reconstituting” OR “fenestrated” OR “reconstituted” AND “bile duct” OR “injury” OR “leak” OR “ERCP” OR “complications” combined with Medical Subject Heading “subtotal laparoscopic surgery”.

Study Selection and Eligible Criteria

This meta-analysis was limited to studies that satisfied the subsequent eligibility requirements: prospective, retrospective, and case-controlled studies comparing fenestrating versus reconstituting subtotal cholecystectomy techniques on patients that reported the outcomes of interest and studies that analyzed both techniques within the same population. We excluded abstracts from conferences, studies that analyzed laparoscopic surgeries with a conversion to open rate exceeding 30%, studies written in a language other than English, studies whose texts were not fully available online, and editorials and review articles.

Screening for post-endoscopic retrograde cholangiopancreatography (post-ERCP), bile leak, and bile duct injury were primary outcomes. Long-term and short-term problems were also examined as secondary results. Furthermore, the subsequent baseline attributes were gathered: number of patients, surgical technique, distribution of sexes, average age, The American Society of Anaesthesiologists (ASA), and procedural indication.

The review protocol was registered at PROSPERO, registration number: CRD42024593070.

Data Extraction and Quality Assessment

Five researchers (C.T., C.K., M.L.J., A.F., S.K.) extracted data based on established criteria, and two senior researchers (K.R. and A.E.) reviewed the results. The data that was retrieved included information on the journal, publication year, databases examined, length of study, number of studies, total patients and nations, study design, outcomes, primary findings, primary limitations, and implications.

Using the Risk of Bias in Non-randomized Studies-of Interventions (ROBINS-I) tool [17], two authors (K.R., A.E.) independently evaluated the quality assessment and risk of bias in each individual study. This is not a randomized study; rather, it is a quantitative tool for estimating the effectiveness of interventions. It assesses bias resulting from confounding, bias in the study's participant selection process, bias in the interventions' classification, bias in the event that intended interventions deviate from reality, bias in the assessment of outcomes, and bias in the choice of the published result. The Risk of Bias Visualization Tool (ROBVIS) was used to construct a quality assessment summary for every publication (ROBVIS) [18].

Statistical Analysis

The Preferred Reporting Items for Systematic Reviews and Meta-Analysis (PRISMA) statement and the Cochrane Collaboration's criteria were followed in the execution of this meta-analysis. With 95% confidence intervals, odds ratios (OR) were used to compare binary endpoints. To pool continuous outcomes, weighted mean differences were applied. Using the Cochran Q test and I2 statistics, heterogeneity was assessed; p-values less than 0.10 and I2 25% would be regarded as significant. Review Manager 5.4 (Nordic Cochrane Centre, The Cochrane Collaboration, Copenhagen, Denmark) was utilized for statistical analysis.

Results

**Figure 1:**
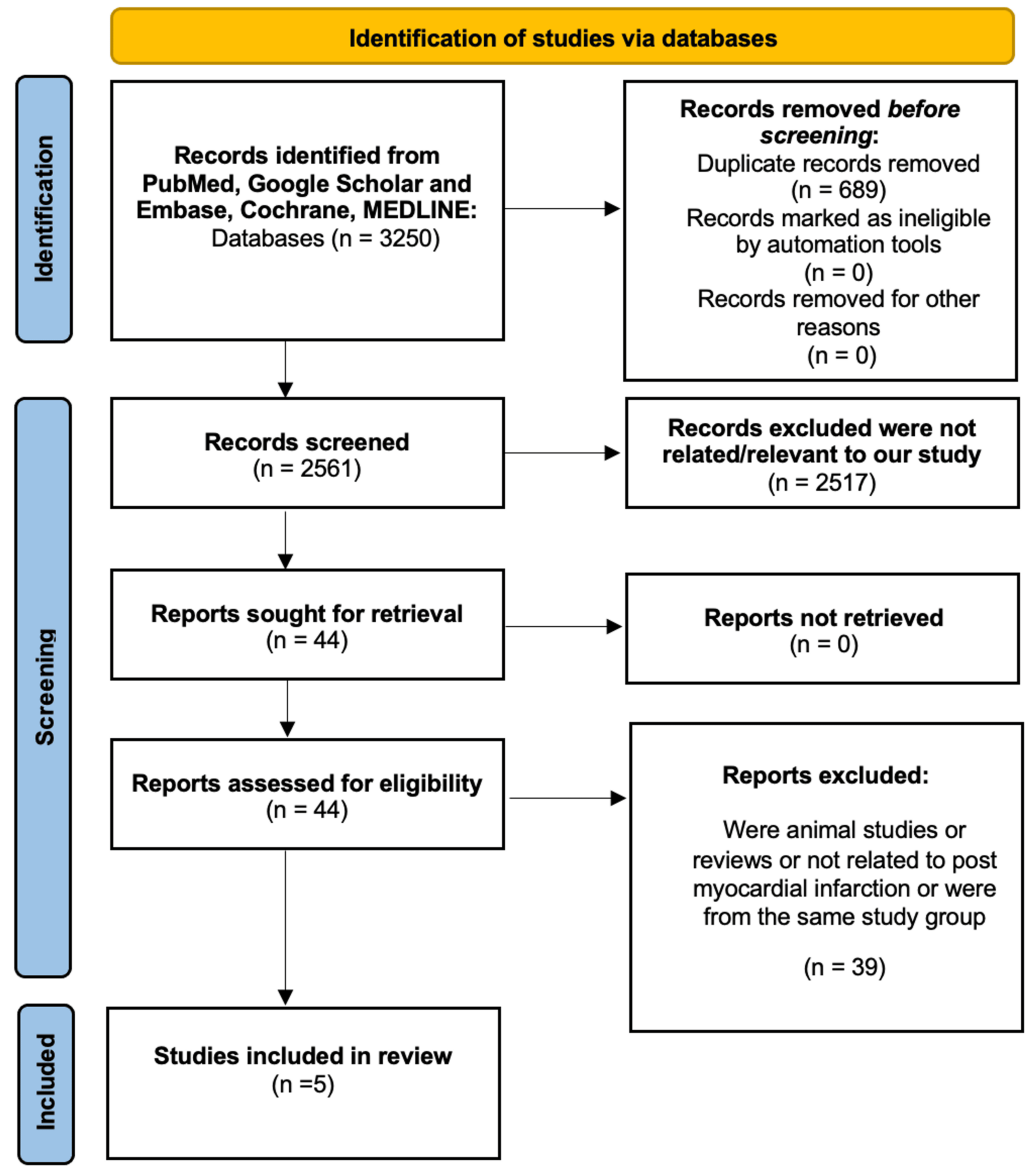
PRISMA flow diagram demonstrating the literature selection strategy PRISMA: Preferred Reporting Items for Systematic Reviews and Meta-analyses

The first database search found 3250 items; we eliminated 689 of them due to duplication. After the exclusion criteria were applied, 2517 of the remainder were eliminated. Thirty-nine papers were eliminated after abstracts were assessed for lack of relevance to the review subject. The final review had five papers in all. Figure 1 displays a PRISMA-style graphic illustrating the book selection process [16].

Among the five studies, 363 (65.8%) had fenestrated subtotal cholecystectomy, while 189 (34.2%) had reconstituted subtotal cholecystectomy [19-23]. The studies included populations from the US, UK, Japan, and Turkey [19-23]. Table 1 summarizes the overall findings from our studies [19-23].

**Table 1:** Summary of publications included in this review R: reconstituted; F: fenestrated

Study	Journal	Type of study	Performed period	Number of patients	Male patients	Mean age (SD)	ASA grade: no of patients (%)	Mean operation time (mins)	Mean hospital stay (days)
Horiuchi et al. [19]	Surgical Endoscopy	Retrospective	2000-2005	R: 3	-	-	-	R: 165	-
				F: 26				F: 133	
Thomas et al. [20]	Surgical Endoscopy	Retrospective	Jan 2015 to Dec 2021	R: 55	R: 26	R: 64 (60, 67)	R: 1 or 2–55 (100%)	R: 107	R: 4
				F: 115	F: 84	F:65 (62, 69)	F: 1 or 2–115 (100%)	F: 91	F: 6
van Dijk et al. [21]	Journal of the American College of Surgeons	Retrospective	May 2005 to Jan 2013	R: 73	R: 32	R: 57.0 (IQR 48.0–66.0)	R: 1 or 2–57 (78.1); 3 or 4–4 (5.5); Unknown–12 (16.4)	R: 136	R: 3
				F: 102	F: 59	F: 65.5 (IQR 50–74)	F: 1 or 2–78 (76.5); 3 or 4–15 (14.7); Unknown–9 (8.8)	F: 107	F: 5
Yildrim et al. [22]	Cureus	Retrospective	Jan 2015 to Dec 2020	R: 34	-	-	-	-	-
				F: 12					
Loh et al. [23]	HPB Journal	Retrospective	Jan 2015 to Dec 2016	R: 24	R: 16	R: 71 (59–83)	R: 1 or 2–16 (66.7) 3 or 4–8 (33.3)	R: 141	R: 6
				F: 108	F: 52	F: 61 (51.5–70.5)	F: 1 or 2–87 (80.6) 3 or 4–21 (19.4)	F: 128	F: 7

Two studies reported the main indication for fenestrated and reconstituted cholecystectomy was acute calculous cholecystitis followed by acute cholangitis [21,23]. A study also reported one incident of myocardial infarction and three mortalities after fenestrated subtotal cholecystectomy [22]. The same study reported two incidents of incisional hernia and one mortality after reconstituted subtotal cholecystectomy [22]. There was also one incident of pancreatitis reported 30 days post-op after fenestrated subtotal cholecystectomy [23].

Bile Duct Injury

**Figure 2:**
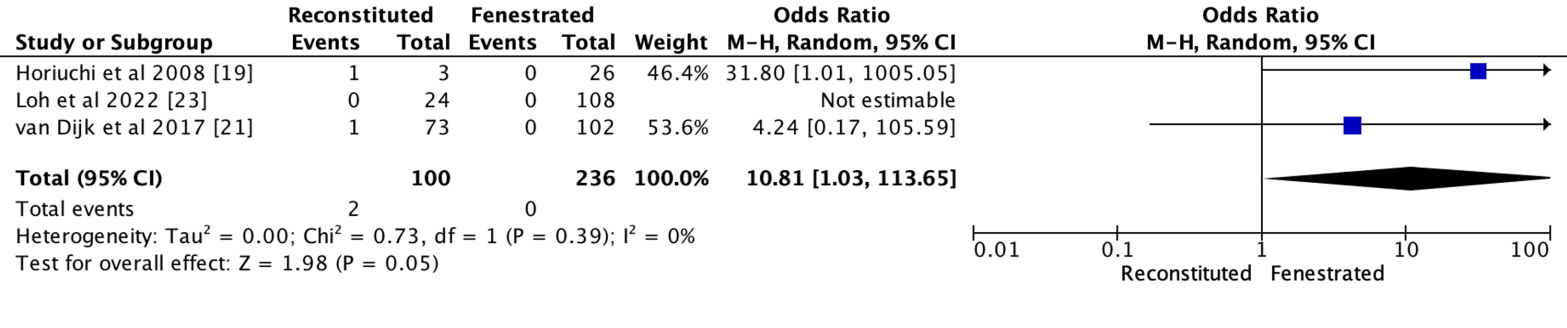
Meta analysis comparing bile duct injury between reconstituted and fenestrated subtotal cholecystectomy

Three studies reported bile duct injury in two patients from three studies [19,21,23]. The reconstituted group reported two bile duct injuries from 100 patients (2%), while the fenestrated group reported no bile injury from 236 patients (0%). The fenestrated group was reported to have a lower incidence than the reconstituted group (OR 10.81; CI 95% 1.03-113.65; p = 0.39; I2 = 0%), as demonstrated in Figure 2.

Bile Leak

**Figure 3:**
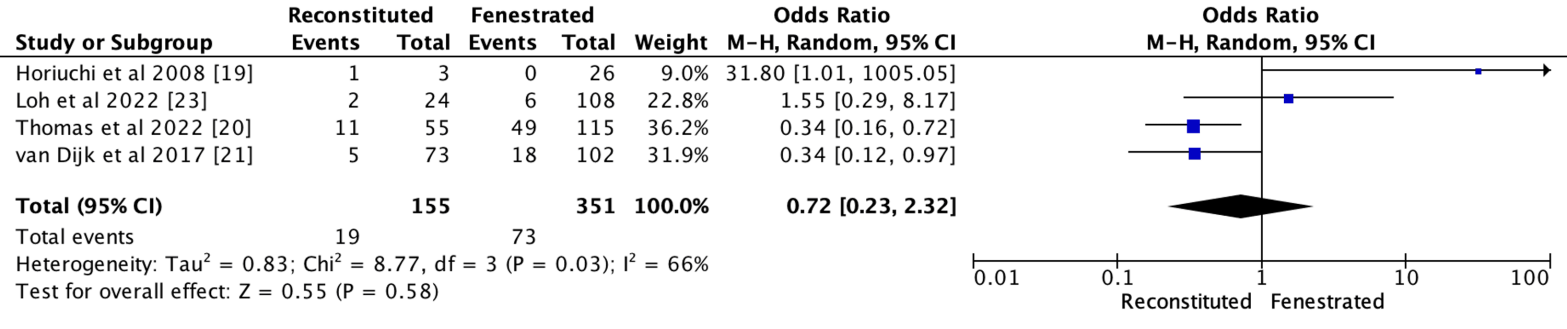
Meta analysis comparing bile leak between reconstituted and fenestrated subtotal cholecystectomy

Bile leaks in 92 individuals were reported in four studies: 19 out of 155 (12.3%) in reconstituted and 73 out of 351 (20.8%) in fenestrated [19-21,23]. As demonstrated in Figure 3, there was a significant difference in bile leak between the two groups (OR 0.72; CI 95% 0.23-2.32; p = 0.03; I2 = 66%).

Fistula Formation Post-operation

**Figure 4:**
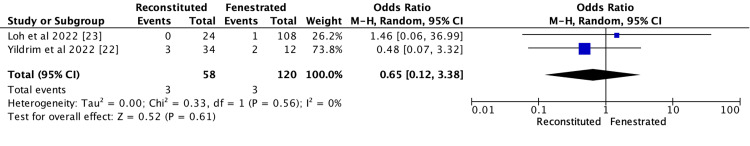
Meta analysis comparing post-operative fistula formation between reconstituted and fenestrated subtotal cholecystectomy

Two studies reported fistula formation post-operation (three out of 58 in the reconstituted group (5.2%) and three out of 120 in the fenestrated group (2.5%)) [22,23]. As demonstrated in Figure 4, there was no significant difference between the groups (OR 0.65; CI 95% 0.12-3.38 p = 0.56; I2 = 0%).

Comparing Whether Post-operative ERCP Was Needed Between the Two Techniques

**Figure 5:**
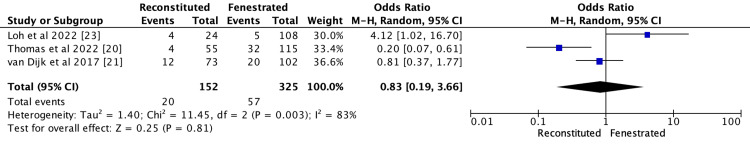
Meta analysis comparing post-ERCP between reconstituted and fenestrated subtotal cholecystectomy

Ninety-seven patients out of 477 patients reported a need for an ERCP after subtotal cholecystectomy from three studies (20 out of 152 in the reconstituted group (13.2%) and 57 out of 352 from the fenestrated group (16.1%)) [20,21,23]. There was a significant difference between the two groups (OR 0.83; CI 95% 0.19-3.66, p = 0.003; I2 = 83%), as demonstrated in Figure 5.

Readmission After Subtotal Cholecystectomy

**Figure 6:**
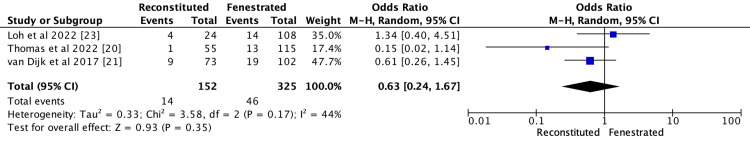
Meta analysis comparing readmission between reconstituted and fenestrated subtotal cholecystectomy

Three studies reported 60 patients had readmission from 477 patients who underwent cholecystectomy (14 out of 152 from the reconstituted group (9.2%) and 46 out of 352 from the fenestrated group (13%)) [20,21,23]. There was no significant difference between the two groups (OR 0.63; CI 95% 0.24-1.67 p = 0.17; I2 = 44%), as demonstrated in Figure 6.

Retained Stones Post Subtotal Cholecystectomy

**Figure 7:**
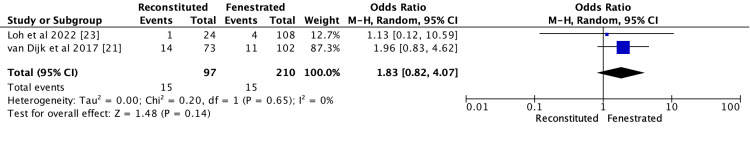
Meta analysis comparing retained stones between reconstituted and fenestrated subtotal cholecystectomy

Thirty patients out of 307 subtotal cholecystectomy patients had retained stones from two studies (the reconstituted group reported 15 out of 97 patients (15.5%), while the fenestrated group reported 15 out of 210 patients (7.1%)) [21,23]. There was no significant difference between the two groups (OR 0.1.83; CI 95% 0.82-4.07 p = 0.65; I2 = 0%), as demonstrated in Figure 7.

Post-operative Drain

**Figure 8:**
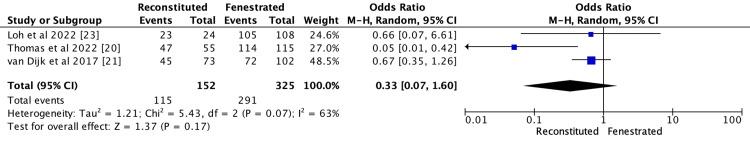
Meta analysis comparing post-operative drain between reconstituted and fenestrated subtotal cholecystectomy

Three studies reported that 442 out of 477 patients required a post-operative drain after subtotal cholecystectomy (reconstituted group: 115 out of 152 (75.7%), fenestrated group: 291 out of 325 (89.5%) [20,21,23]. The reconstituted group reported a lower incidence than the fenestrated group (OR 0.33; CI 95% 0.07-1.60; p = 0.07; I2 = 63%), as demonstrated in Figure 8.

Wound Infection Post Subtotal Cholecystectomy

**Figure 9:**
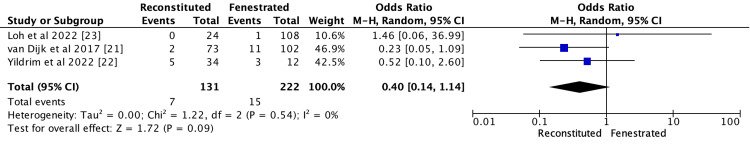
Meta analysis comparing post-operative wound infection between reconstituted and fenestrated subtotal cholecystectomy

Twenty-two patients from 353 patients reported to have had wound infection after subtotal cholecystectomy in three studies (reconstituted group: 7/131 (5.3%), fenestrated group: 15/222 (6.8%)) [21-23]. The reconstituted group reported a lower incidence rate than the fenestrated group (OR 0.40; CI 95% 0.14-1.14; p = 0.54; I2 = 0%), as demonstrated in Figure 9.

ASA Grade 1-2

**Figure 10:**
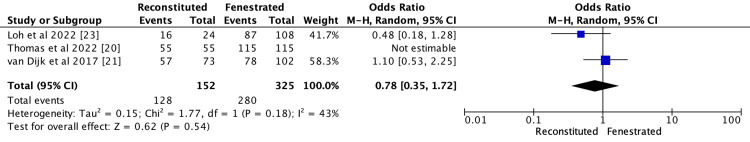
Meta analysis comparing ASA grade 1-2 between reconstituted and fenestrated subtotal cholecystectomy

Three hundred and eight out of 477 patients were reported to have an ASA grade of 1 of 2 from three studies (128 out of 152 in the reconstituted group (84.2%), 280 out of 325 in the fenestrated group (86.2%)) [20,21,23]. There was no significant difference between the groups (OR 0.78; CI 95% 0.35-1.72; p = 0.18; I2 = 43%), as demonstrated in Figure 10.

Quality Assessment

**Figure 11:**
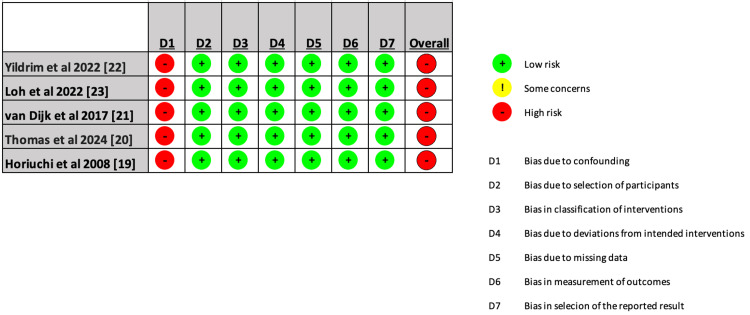
Detailed assessment of every study included in the systematic review using the ROBVIS ROBVIS: risk of bias visualization tool [12]; D1: bias due to confounding; D2: bias due to selection of participants; D3: bias in classification of interventions; D4: bias due to deviations from intended interventions; D5: bias due to missing data; D6: bias in measurement of outcomes; D7: bias in selection of the reported result

The included studies were categorized as having a significant risk of bias overall. Due to confounding, the majority of studies were deemed to have a severe risk of bias since they are retrospective chart review studies. Figure 11 displays an individual evaluation of each study that was a part of the meta-analysis.

Discussion

The laparoscopic cholecystectomy has become incredibly popular among surgeons and is a prime example of a change in worldwide practice that didn't require a randomized study [24]. An increased risk of bile duct injury was caused by inexperience, a lack of systematic training, and incorrect interpretation of the laparoscopy picture [24]. Although the frequency of BDI has peaked, it cannot be completely eradicated even when formal training and the learning curve have been surpassed [24]. Numerous techniques are employed to reduce the risk of complications during laparoscopic cholecystectomy [24]. Among these is subtotal cholecystectomy [24]. The reported prevalence of common bile duct injuries in all subtotal cholecystectomies ranges from 0.5% to 0.2, indicating the potential efficacy of subtotal cholecystectomy in averting common bile duct injuries [25]. Our review and studies reported similar findings. This demonstrates how successful subtotal cholecystectomy may be in avoiding typical bile duct damage [26].

Subtotal cholecystectomy proportions can reach up to 10% of all cholecystectomies; thus, surgeons must have a thorough understanding of the issue in order to conduct this treatment as well as feasible [27-30]. Before the treatment, it is critical to assess the risk of a challenging cholecystectomy that might progress to a subtotal cholecystectomy [31,32]. This can be done by establishing a suitable timetable, contacting family members, and seeking assistance [31,32]. As it depends on several factors rather than just one, various scores have been developed to help predict this risk and may be a better indicator of a difficult cholecystectomy [31,32].

Bile leak is one of the most common problems; thus, the best option for preventing it when subtotal closed-tract cholecystectomy is not an option appears to be internal suture-closure of the cystic duct or an alternate procedure such as an omental plugging or falciform ligament patch [33-35]. More research is necessary to assess the efficacy of omental plugging and the falciform ligament patch, though, as there is a dearth of information on these procedures in the literature [33-35]. The blockage of the cystic duct with cyanoacrylate glue is another method for preventing bile leaks; however, this operation is not advised as the glue might migrate to the bile duct and induce bile duct obstruction [33-35]. Our study showed fenestrated had a higher risk of bile leak than reconstituted (20.8% vs. 12.3%, respectively). This result is probably due to technical issues with the fenestrating procedure, which does not need cystic duct closure or entail shutting the residual gallbladder [26].

The question of whether ERCP is always required to treat bile leakage after subtotal cholecystectomy is still up for debate, and some surgeons support monitoring if an intraoperative drain is implanted and the output is poor [36]. To speed up surgical recovery and reduce morbidity related to this problem, the majority do, however, support early ERCP and biliary decompression [26]. It is also critical to emphasize that some individuals may not be candidates for ERCP and that consequences from an extra operation might include bleeding, perforation, and pancreatitis [26]. In the reviewed studies, there were inconsistent indications for ERCP and no information about the time to ERCP or biliary drain output.

The necessity of a subsequent complete cholecystectomy is a crucial factor to consider when considering subtotal cholecystectomy [37]. It is hypothesized that suturing the remaining gallbladder in the reconstituting technique increases the likelihood of new gallstone formation and the requirement for full cholecystectomy [37]. This could be a reason for readmission and complications post-subtotal cholecystectomy.

Limitations

Due to the nature of retrospective data collection, the majority of the included research may have biases due to many of the reviewed studies being retrospective. Therefore, further randomized prospective studies would be recommended for further analysis of the results obtained and to clarify results found from the studies analyzed. Furthermore, it is crucial to emphasize that variations exist in the research methodologies, namely in the execution of cystic duct closure and the absence of the fenestrated technique, which may influence our results. Furthermore, the absence of defined indications for ERCP after fenestrated STC may have contributed to higher rates of ERCPs and needless treatments for some bile leaks that may have been mended on their own. Longer follow-up and more precise technical information should be provided by future research to enable more thorough comparisons on this crucial subject.

## Conclusions

When a difficult laparoscopic cholecystectomy cannot be performed because of a lack of safety, insufficient identification of the anatomical structures involved, or a danger of harm, a surgical bail-out treatment known as subtotal cholecystectomy is recommended. A fenestrating procedure leaves the remaining portion of the gallbladder exposed, and a purse-string suture can seal the cystic duct. The reconstituting procedure effectively creates a residual gallbladder by using sutures or staples to seal the remaining portion of the gallbladder. Post-operative bile leakage is more common following fenestrated partial cholecystectomy, along with fistula formation, wound infection, and retained stones. Our study revealed that bile duct leak was less common in reconstituted procedures, while bile duct injury was less common in fenestrated procedures. We also observed a greater frequency of postoperative ERCP using the fenestrated approach. Longer-term follow-up studies, such as randomized prospective studies and larger national studies, are still required to fully comprehend the place of each approach in the general surgeon's toolbox for treating challenging gallbladder cases.
